# The portrayal of electronic cigarettes in Indonesia: a content analysis of news media

**DOI:** 10.1186/s12889-022-14886-z

**Published:** 2023-01-07

**Authors:** Mouhamad Bigwanto, Imas Arumsari, Ridhwan Fauzi

**Affiliations:** 1 Doctoral School of Psychology, Eötvös Loránd University, Izabella U. 46, Budapest, 1064 Hungary; 2 Institute of Psychology, Eötvös Loránd University, Izabella U. 46, Budapest, 1064 Hungary; 3 Faculty of Health Sciences, Universitas Muhammadiyah Prof. Dr. HAMKA, Jl. Limau II, Jakarta, 12210 Indonesia; 4 Faculty of Public Health, Universitas Muhammadiyah Jakarta, Jl. KH Ahmad Dahlan, Jakarta, 15419 Indonesia

**Keywords:** e-cigarettes, Policy, Advocacy, News media, Content analysis, Indonesia

## Abstract

**Background:**

The news media play an essential role in shaping public opinion. Analyzing a specific issue reported in the news media provides insight and considerations for a government to form a policy. This study aimed to assess the portrayal of electronic cigarettes (e-cigarettes) in Indonesian news media, including the variety of arguments being presented and the actors behind them.

**Methods:**

We used a paid service database from eBdesk to collect Indonesian news articles from 2020 to 2021 using the keywords ‘rokok elektronik’ (electronic cigarette), ‘rokok elektrik’ (electric cigarette), ‘e-rokok’ (e-cigarette), ‘vape’, and ‘vaping’. Content analysis of 551 full-text news articles was conducted to identify the concepts, topics, and frames of quoted arguments and to examine whether any frames were presented by different types of organizations and the origin of actors.

**Results:**

News articles related to e-cigarettes were mostly published in the national newspaper and in the non-health section of the newspaper desk. A total of 891 arguments from 393 persons representing 212 institutions were identified. Twenty-eight concepts were further categorized into 12 topics. Health impact was the most frequently reported topic, followed by regulation, tax/price, and e-cigarettes as smoking cessation tools. Overall, the articles and arguments with positive tones toward e-cigarettes outnumbered negative articles and arguments. The industry, university, and research-based institutions were the most involved types of organizations in the news articles. There were more neutral frames toward e-cigarettes among government, even though the frame within the non-health government sector was reported to be more positive toward e-cigarettes.

**Conclusions:**

Although health impact is the most reported topic, the actors involved in delivering arguments were mainly from the non-health sector, including when the news articles were published by the newspaper desk. The e-cigarette issue in Indonesia was mainly played by the non-health sector, which is more inclined toward economic interest than health.

## Background

Tobacco kills more than 8 million people every year, most of them occurring in low- and middle-income countries [1]. Indonesia, a non-party country to the World Health Organization (WHO) Framework Convention on Tobacco Control, is home to approximately 64.9 million smokers, making it the third highest number of smokers in the world after China and India. Approximately two out of three adult men in Indonesia smoke. The prevalence of smokers (male and female) increased from 32.8% in 2016 to 33.8% in 2018; this increase was due to the doubling of female smokers from 2.5 to 4.8% [2]. While low- and middle-income countries are experiencing difficulties in reducing the prevalence of smoking, including in Indonesia, the industry is now promoting new tobacco products, one of which is e-cigarettes [3]. E-cigarettes have been promoted and marketed as less harmful [4]. However, the WHO report in 2020 clearly stated that these products are undoubtedly harmful to human health [5].

Instead of being a solution, e-cigarette use among youth has increased dramatically [3, 6]. The products have been reported to be available in various flavors and packaging and are promoted by taking advantage of new media [7–9]. A previous study on e-cigarette promotional content on Instagram among 36 different countries found that e-cigarette posts were mostly made in the US (38.5%), Indonesia (14.6%), and the United Kingdom (9.3%) [10], and they were designed to attract youths’ attention [11]. The fact that social media is being used to promote e-cigarette use is also supported by previous systematic reviews analyzing the effect of social media interaction on both intention and current use of e-cigarettes [1]. Specifically, celebrity endorsement on Instagram increased intention to use e-cigarettes [2]. The role of social media in influencing e-cigarette use among youth was also confirmed on other social media platforms, such as Facebook and YouTube [3–5].

Although the media is now changing and developing rapidly, the news media still seems to have a significant place, especially in the political process [12]. The role of the news media in changing public opinion is significant; therefore, the analysis of what is reported in news media regarding a specific issue can provide insight into what people think [13]. All discourse and opinion debates that grow in the news media will also be one of the considerations for a government to form a policy [14]. Currently, there are differences in how countries in the Association of Southeast Asian Nations regulate e-cigarettes. Five countries—Brunei, Cambodia, Thailand, Singapore, and Laos—have totally banned e-cigarette products. Myanmar and Vietnam reported having no specific regulations for the products, while Indonesia, Malaysia, and the Philippines permit the sale of e-cigarettes with certain restrictions applied (control of the products) [15]. However, the only regulation that exists in Indonesia on e-cigarette products is the excise tax imposition [16], which makes the control of the products in the country very weak, including their media promotion.

As there are no non-fiscal regulations for the products in Indonesia, the news media will probably become a battleground to achieve goals that benefit certain parties. Therefore, the effort to monitor the news media for tobacco control measures could not be ignored [17, 18]. Previous research has analyzed the content of e-cigarette topics in Indonesian news articles. However, the studies were limited only to the topics of regulations and harm reduction and were only conducted in four news media. The previous studies also did not identify the type of organization or actor behind all delivered arguments in the news article [19, 20]. Therefore, the full picture of e-cigarette content in Indonesian news media might not have been captured. To complement prior research, this study aimed to assess the portrayal of e-cigarettes in the news media, including the variety of arguments presented and the actors behind those arguments.

## Methods

### Sample

This study integrated the methodology used in previous studies by Lyu, Yates, Wackowski, and Kim [18, 21–23]. To collect Indonesian news articles from 2020 to 2021 (January 1 to December 31), we used a paid service database from eBdesk, which is a research-based company focused on information management implementation using artificial intelligence [24]. We used the keywords ‘rokok elektronik’ (electronic cigarette), ‘rokok elektrik’ (electric cigarette), ‘e-rokok’ (e-cigarette), ‘vape’, and ‘vaping’. Based on that requirement, we identified a total of 1,264 news articles from both online news services and newspapers.

We removed all duplicated and sponsored news articles that intended to promote a specific store or brand. We also removed all news articles that did not contain a statement, which is in line with the aim of the study (to capture a variety of arguments presented in the news media and the actors or persons behind those arguments). A total of 713 news articles were excluded from the analysis. This study did not include other forms of media, such as blogs, journals, and magazines.

In this study, the information collected included newspaper name, section category of the newspaper desk (health or non-health), date, newspaper type (national or local), name of the person (actor) who conveyed the argument, and the origin of the organization (domestic or international). Newspapers that use the name of the city or province in Indonesia (e.g., *Tribun News Bali*) were categorized as non-national newspapers.

### Content coding

Three persons collected all the information and coded the news articles using Microsoft Excel. One coder represented the author, while others were paid, trained coders. The first coder was responsible for collecting and coding all news articles published from January 1^st^ to March 31^st^ 2020, the second coder from April 1^st^ 2020 to March 31^st^ 2021, and the last coder from April 1^st^ to December 31^st^ 2021.

A total of 75 news articles (13.6% of the sample) were read to test and refine the codebook by the first author. Afterwards, a total of 30% (*n* = 164) of the news articles were tested and triple-coded. The four variables included in the triple coding were: 1) topics, 2) frames, 3) type of organization, and 4) the origin of actors or the organization. The details about triple-coded results are available in the reliability test section.

We defined an argument as a full-quoted statement delivered in the news article by a person, while an actor is a person who delivered the statement. For example, the news article titled ‘Strong Regulations Need to Save Children from E-Cigarette Bondage’ was published in *Jurnal Nasional* (*Jurnas*) on 26^th^ June 2020. The news article quoted statements from Mouhamad Bigwanto from the Southeast Asia Tobacco Control Alliance (SEATCA): *“It is important for the government to immediately make regulations to regulate the circulation and promotion of electronic cigarettes.…Don’t let our children and youth be made easy targets again by the industry”.* The article also published another argument from Lisda Sundari from the Lentera Anak Foundation: *“Because of this ignorance, not a few parents swallow the information that electronic cigarettes are safe and can help people quit smoking….Strong regulation is needed to protect Indonesia from the negative effects of electronic cigarettes”.*

From the above example, we first categorized the newspaper under the national category with no information about the section or news desk. Bigwanto and Sundari were the actors from an international and domestic organization. We also categorized the quoted arguments above as negative frames toward e-cigarettes. Frame refers to the positioning or perspective of each argument regarding e-cigarettes delivered by a person in the news article. Depending on how many people and arguments were delivered in the news articles, one news article could have more than one frame. While argument is a full-quoted statement from a person in the news article, a concept is the generic idea generalized from those arguments. A total of 28 concepts were identified in this study. For instance, a full-quoted argument from the above example has a generic concept that asks for strict regulation of e-cigarettes.

Besides categorizing concepts, we also grouped all concepts into several topics. From those 28 identified concepts, all authors, together with the coders, had a discussion to determine the topics of those concepts. For example, the arguments, “*there is no doubt that they are (e-cigarettes) harmful to health and are not safe*” and “*in principle, all types of cigarettes or tobacco are harmful to health*”, were both categorized in the same concept: e-cigarettes are harmful to health. The concept ‘e-cigarettes are harmful to health’ was then coded as a ‘health impact’ topic. Another example is the concept ‘e-cigarettes should be strictly regulated’ from the previous example coded as a ‘regulation’ topic. After the discussion, a total of 12 topics from 28 concepts were identified.

Last, we categorized the overall news article’s tone from the frames of argument presented within it. The tone was the perspective that had been described in the whole article. An article could only have one tone (positive, negative, or neutral). Only an article with a balanced perspective was categorized as neutral.

**Fig. 1:**
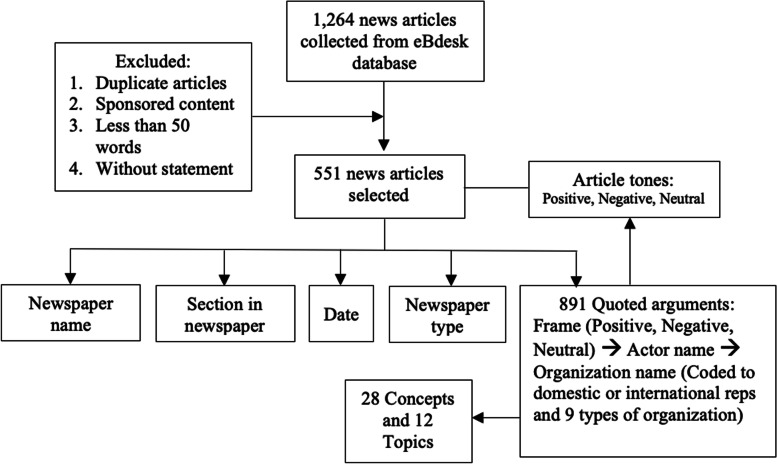
Study design flow chart

Coding was also conducted on the type of organization of the actor in the article. The actor was categorized as domestic or international representatives, and the organizational type was divided into nine categories: 1) university, 2) industry association, 3) consumer groups, 4) health groups, 5) research groups, 6) farmers, 7) religious groups, 8) other types of non-government organizations (NGOs), and 9) government organizations. In the final analysis, we recategorized the NGOs into university and research-based institutions, health groups (health professional organizations), and other types of NGOs. Government organization was divided into health sector (the Ministry of Health and Food and Drug Administration) or non-health sector (Fig. 1).

### Reliability test

Before coding, a total of 30% (*n* = 164) of the news articles were randomly selected and triple-coded to ensure that the coding processes among the three coders were reliable. The data were examined using Krippendorff’s alpha inter-coder reliability test. The results of the test ranged from 0.87 to 0.92.

## Results

A total of 551 news articles from 128 newspapers were included in the analysis, of which 44.5% (*n* = 57) were national newspapers (*Tempo*, *Kompas*, *The Jakarta Post*, *Antara*, *Republika*, *Sindo*, *Detik.com*, etc.). News articles related to e-cigarettes were mostly published in national newspapers (73.3%) and in the non-health section of the newspaper desk (86.4%). More than half of the news articles (*n* = 303, 55%) had a favorable tone toward e-cigarettes (Table 1).

**Table 1:** Newspaper type, section, and article tones by year

Year (*N* = 551)	Newspaper Type (%)		Newspaper Section (%)		Article Tones (%)		
	National	Sub-national	Non-health	Health	Positive toward e-cig	Negative toward e-cig	Neutral
2020 (*n* = 312, 56.6%)	196	64	178	41	146	94	20
2021 (*n* = 239, 43.4%)	208	83	298	34	157	66	68
	**404 (73.3)**	147 (26.7)	**476 (86.4)**	75 (13.6)	**303 (55)**	160 (29)	88 (16)

A total of 891 arguments from 551 news articles were identified. These arguments were quoted from 393 persons representing 212 institutions. More than two-thirds (*n* = 609, 68.3%) of the arguments were framed as positive toward e-cigarettes, followed by negative (*n* = 245, 27.5%) and neutral frames (*n* = 37, 4.2%). The arguments mostly (86.9%) came from NGOs and were delivered by domestic or local actors or organizations (75.8%). Regarding statements from government institutions, more than half (74.4%) were delivered by the non-health sector. There were more neutral frames toward e-cigarettes within government sector, even though the frame within the non-health government sector was reported to be more positive toward e-cigarettes (Table 2).

**Table 2:** Statement frames by type of organization, origin of actors, and type of government

	Statement frames			*P*-value
	Positive toward e-cig	Neutral	Negative toward e-cig	
Type of organization (*N* = 891)				
Government (*n* = 117, 13.1%)	50 (42.7%)	22 (18.8%)	45 (38.5%)	0.000
NGOs (*n* = 774, 86.9%)	559 (72.2%)	15 (1.9%)	200 (25.8%)	
Origin of actors (*N* = 891)				
Domestic (*n* = 675, 75.8%)	500 (74.1%)	31 (4.6%)	144 (21.3%)	0.000
International (*n* = 216, 24.2%)	109 (50.5%)	6 (2.8%)	101 (46.8%)	
Type of government (*N* = 117)				
Non-health (*n* = 87, 74.4%)	43 (49.4%)	17 (19.5%)	27 (31%)	0.014
Health (*n* = 30, 25.6%)	7 (23.3%)	5 (16.7%)	18 (60%)	

Health impact was the most frequent topic (29.8%), followed by regulation (18.9%), tax/price (9.8%), and e-cigarettes as smoking cessation tools (8.9%). The only non-conflicting concept in the media was about the environment, in which e-cigarettes were framed positively for the environment. The voices from the industry almost outnumber voices from the NGOs combined (out of university, research-based organizations, and health groups). There were only two statements from the industry group that were unfavorable toward e-cigarettes on the topic of regulation and the consequences of e-cigarette use.

We found that the types of NGO that promoted e-cigarettes varied and could be categorized into several groups: 1) the industry and its associations (e.g., the Association of Indonesian Nicotine Delivery Entrepreneurs and the Indonesia Vapor Entrepreneurs Association), 2) university and research-based organizations (e.g., the Center for Youth and Population Research and the Institute for Development of Economics and Finance), 3) health groups (e.g., the Indonesian Young Pharmacist Group), and 4) other types of NGOs such as faith-based organizations (e.g., PBNU’s Institute for Research and Human Resources Development/Lakpesdam PBNU), consumer groups (e.g., Organized Vape Consumers and the Indonesian Vape Consumer Association) and coalition groups, such as the Indonesia Tar-Free Coalition, which was founded by the Public Health Observer Foundation.

Voices from the university and research-based organizations were reported at 69.8% (*n* = 150) in favor of e-cigarettes on all topics. They represented 20.9% (*n* = 115) of the news articles, with a total of 215 (24.1%) arguments delivered. A contrasting situation was shown in health groups, except for the topic of the consequences of e-cigarettes, which several health groups and organizations believe could help the government to reduce smoking prevalence. On the other hand, the government showed more neutral voices than other organizations, especially on topics like regulation, tax/price, and the national standard certification (SNI) for e-cigarette products (Table 3).

**Table 3:** Topics and conflicting concepts by the type of organization

**Topics and (conflicting) Concepts**	**Type of organizations**				
	Non-Government Organizations				Govt
	Industry	Univ & Research-based	Health Groups	Other NGOs	
**Health Impacts of e-cigarettes (n = 266, 29.8%)**					
Harmful ***(n* = *137)***	-	41	40	43	13
Not/less harmful (*n* = 125)	25	53	6	37	4
The health impact still not yet concluded (*n* = 4)	-	2	-	2	-
**Regulation (*n* = 168, 18.9%)**					
Should be banned/strictly regulated (*n* = 38)	1	3	7	13	14
Need special regulation (less strict than cigarettes), banning will only put smokers in danger and increase illegal products ***(n* = *123)***	43	33	1	38	8
Still need more considerations (*n* = 7)	-	1	-	-	6
**Tax or Price (*n* = 87, 9.8%)**					
Need to raise its tax to control the consumption, especially among youth (*n* = 8)	-	1	-	2	5
E-cigarette price is too expensive, need an incentive (tax reduction) for low-risk products and to accelerate the investment ***(n* = *70)***	40	12	-	12	6
Need further discussion to determine the tax policy (*n* = 9)	-	-	-	-	9
**Cessation (*n* = 79, 8.9%)**					
E-cigarette is not an effective tool for smoking cessation (*n* = 15)	-	4	4	4	3
E-cigarette is an effective tool for smoking cessation ***(n* = *62)***	16	19	3	20	4
Still need more study (*n* = 2)	-	1	-	1	-
**Persuasive message to government (*n* = 69, 7.7%)**					
Protect future generation from nicotine addiction (*n* = 7)	-	-	-	7	-
Government was asked to be open to alternative tobacco products, and the industry is ready to collaborate with the government to prevent youth use of e-cigarettes ***(n* = *62)***	22	14	-	24	2
**Economy (*n* = 56, 6.3%)**					
Need to consider the increase in health costs (*n* = 3)	-	-	1	1	1
Create jobs, increase state and economic income, good for tobacco farmers’ welfare ***(n* = *53)***	38	2	-	5	8
**The Indonesian National Standard Certification (SNI) for e-cigarettes (*n* = 39, 4.4%)**					
Given SNI for a harmful product is wrong and will only benefit the industry (*n* = 5)	-	-	1	4	-
SNI needed to protect e-cigarette customers and increase public confidence ***(n* = *31)***	12	3	-	2	14
Need more time and consideration (*n* = 3)	-	-	-	-	3
**Consumers (*n* = 38, 4.3%)**					
Customers (youth) need to be protected from misleading (less or harmless) information about e-cigarettes and to prevent e-cigarettes as a gateway to smoking (*n* = 10)	-	2	3	3	2
Consumers have the right to choose low-risk products ***(n* = *27)***	10	6	-	9	2
Should be more careful in summarizing research results (*n* = 1)	-	-	-	-	1
**E-cigarette consequences (*n* = 33, 3.7%)**					
Cause a double burden (both smoking and e-cigarette use prevalence increased) (*n* = 11)	1	3	1	4	2
Could help government to reduce smoking prevalence ***(n* = *22)***	4	8	5	4	1
**Religious issue of e-cigarettes (*n* = 11, 1.2%)**					
E-cigarettes are a haram product (prohibited in Islam) (*n* = 3)	-	-	-	3	-
E-cigarettes are not a haram product ***(n* = *5)***	1	-	-	4	-
Still need more study and discussion (*n* = 3)	-	-	-	3	-
**Environmental (*n* = 2, 0.2%)**					
The use of alternative tobacco products can reduce the danger of polluting the environment	-	-	-	2	-
**Others (*n* = 43, 4.8%)**	19	7	-	8	9
**Total**	232	215	72	255	117

Among all topics, the concept of e-cigarettes being harmful to health is the only negative frame that outnumbered the positive one. However, the number of positive frames toward e-cigarettes in this topic was higher than in other topics. Regarding regulation, both concepts (positive and negative frames toward e-cigarettes) were actually calling for the same objective: to regulate the products. However, the degree of regulation being asked was different; the negative frame asked for banning regulation or at least strict regulation to control consumption, while the positive one asked for special regulation, different, or more relaxed from the current regulation for combustible cigarettes.

On the topic of tax, we found that the COVID-19 situation has been used to pressure the government to give incentives to the industry (not to raise tax for e-cigarettes) to boost the economy after the pandemic.

*“The high excise tax on the liquid closed system has made the industry players feel even more severe. The volume of closed-system vaporizer users has decreased by around 60-70 percent due to the COVID-19 pandemic. Therefore, we really hope that there will be a review, otherwise it will be difficult for us to survive.”* Association of Indonesian Nicotine Delivery Entrepreneurs, Bisnis.com, Sept 17^th^, 2020.

The cessation topic was dominated by the positive frame toward e-cigarettes; only a few counter-arguments were presented on this topic. On the other hand, both positive and negative frames toward e-cigarettes used child protection arguments as persuasive messages to the government. For example, the statement from the Indonesia Vapers Association claimed that they are ready to collaborate with the government to prevent minors from using e-cigarettes.

*“All parties must play an active role so that opportunities to access alternative tobacco products are not opened to children under the age of 18 and those who do not smoke.”* AVI, Pikiran Rakyat, June 1st, 2020.

Another persuasive message being used is to conduct research and be open to innovations (products):

*“We openly invite key stakeholders, such as the Government through the National Research and Innovation Agency (BRIN) and also the Ministry of Health and industry players to be able to conduct more in-depth studies (on e-cigarettes) in the country,”* Public Health Observer Foundation (YPKP), Suara.com, November 10^th^, 2020.

The positive frames toward e-cigarettes also use economic arguments, such as creating new jobs and increasing government income from taxes. E-cigarettes are also being framed positively for tobacco farmers’ welfare. On the other hand, the possible increase in health costs is still the main argument for the negative frames toward e-cigarettes.

*“This electric cigarette uses tobacco products from farmers. Tobacco, which is not completely absorbed by the cigarette industry, can be used in liquid vape”*, the Indonesia Vapor Entrepreneurs Association (APVI), Suara Merdeka, January 15th, 2020.

On the topic of consumers, the right to access low-risk products has become the main argument for the positive frames. Moreover, consumer protection has also been used to push or cancel the government plan in developing the Indonesian National Standard Certification (SNI) for e-cigarette products. E-cigarettes were also framed positively as the solution to reduce smoking prevalence.

The last conflicting concept is about the haram fatwa of e-cigarettes (or the forbidden use based on the interpretation of Islamic law given by a qualified scholar), which involved faith-based organizations. On the topic of the environment, the only concept that appeared was the positive frame toward e-cigarettes, which claimed that they could reduce the danger to the environment.

## Discussion

This study reported that positive articles and arguments about e-cigarettes in Indonesia outnumbered the negative articles and arguments. Health impact, regulation, tax, and price were the most dominant topics presented in the news media. On the topic of health impact, this study strengthens the findings from a previous study in Indonesia where the negative frame toward e-cigarettes (e-cigarettes are harmful) was reported to be more dominant compared to the positive frame (e-cigarettes are not/less harmful) [19]. However, despite the dominance of negative frames, the number of positive frames toward e-cigarettes on the health-impact topic was higher than on other topics. This indicates how massive the harm reduction frames were in the news media and shows the importance of health impacts as a key in influencing public opinion and the government in forming a policy [21, 25], besides directly discussing regulations for e-cigarettes in the news media [18, 21, 23, 26]. In the past, the tobacco industry did the same thing for light and mild cigarette products, which were promoted as low-risk products (low tar). As a result, smokers have been reported to tend to believe these claims [27].

Despite the high number of health-impact arguments in the news media, the content on social media promotion appears to be different. A recent study of e-cigarette promotional content on Instagram in Indonesia showed that only 13% of content uses health impact (less harmful) as their promotional content. Most content features women’s image models and highlights the use of the product and flavor [28]. The different characteristics between news and social media might be the answer to this different phenomenon, where social media, especially Instagram, is more depicted in images or graphic information.

Besides the harm reduction argument, the study also shows how the industry has persistently been pushing for special regulations for e-cigarettes, different from conventional cigarettes, due to the claim that e-cigarettes are less harmful. When the Ministry of Finance decided to implement the excise tax imposition on e-cigarettes in 2018, this policy was regarded as the first regulation for the products and made e-cigarettes officially legal in Indonesia. Following that decision, on 18 July 2018, the Ministry of Finance disseminated the first Excisable Goods Entrepreneur Identification Number (NPPBKC) to all registered e-cigarette entrepreneurs in Indonesia [29]. Since then, the industry has declared July 18 as National Vape Day in Indonesia [30]. This shows how important the 2018 tax-imposed decision was for the industry.

The industry, university, and research-based institutions were the most involved types of organizations in the news articles. Voices from the university and research-based organizations reported in favor of e-cigarettes on all topics. This reminds us of the tobacco industry’s long history of involving researchers to support its capital interest. One of the strategies is to produce doubt about the harmful effect on health in the media and keep the controversy alive [31]. Evidence has been exposed on how the tobacco industry produced misleading information, from providing $45 million in funds to United States scientists from 1979 to 1985 to generating biomedical research evidence in favor of the industry to attacking the government agency about secondhand smoke risks in the mid-1990s [32]. The latest case is how JUUL sponsors special edition articles to be published in one of the United States scientific journals [33].

Almost all major multi-national tobacco industries have produced and marketed new tobacco products, some of which have been marketed in Southeast Asian countries [34]. Although the number of e-cigarette users has been growing, the market for e-cigarettes in Indonesia is relatively small compared to combustible cigarettes [35–37]. Therefore, the main domestic tobacco industry is still not involved in the e-cigarette business. This explains why there were unfavorable statements from the industry toward e-cigarettes in this study. Some tobacco industries feel they are competing with this new product [38], while others see this as an opportunity, so they continue supporting e-cigarette industries and their subsidiaries. For instance, PT HM Sampoerna, a subsidiary of Philip Morris International in Indonesia, has funded the Indonesia Tar-Free Coalition (KABAR) [39]. This coalition was formed by the Public Health Observer Foundation (YPKP), which has been the leading front group promoting the new nicotine tobacco products since 2016 [39]. PT HM Sampoerna is also reported to support the Indonesia Vapor Entrepreneurs Association (APVI), a major e-cigarette industry association in Indonesia [40].

This tactic is similar to those successfully applied to cigarette tax regulations in 2018. The tobacco industry has successfully mobilized front groups and disseminated misleading evidence using news media to undermine tobacco tax regulation in Indonesia [41]. The topic of tax and price is also reported as the third most frequently discussed topic in the news media. There were conflicting concepts on tax and price in this report, where the positive concept toward e-cigarettes is about the economic recovery, investment, and incentives for so-called low-risk products. According to Excise Law No. 39/2007, the imposition of the excise tax is primarily intended to control consumption. However, since the excise tax implementation for e-cigarettes in 2018, there has been no other regulation that could limit consumption, including protecting minors where the prevalence of e-cigarette use among youth aged 10–18 years increased dramatically from 1.2% in 2016 to 10.9% in 2018, much higher than the prevalence for adults in the same period (2 to 2.7%) [37, 42].

Although the 2018 tax-imposed regulation was regarded as one of the biggest wins for the industry, pushing lower tax and price is still the main agenda in the news media. We also found that the COVID-19 situation has been used to pressure the government to provide incentives to the industry. During this study period, several tax incentives were given by the government, including the decision to ease excise payment for the tobacco industry by extending the payment deadline from 60 to 90 days [43].

Along with the argument of e-cigarettes as an effective tool for smoking cessation, the argument that e-cigarettes could reduce smoking prevalence has also been used to promote these products to the government. The industry is trying to influence and convince policymakers that they are ‘transforming’ and could be part of the solution to the smoking problem, including how they are willing to collaborate with the government in preventing child tobacco use. A recent study evaluated this ‘transformation’ claimed by the industry, and the result was that no tobacco company met the criteria of ‘transformation’. The industry is still trying to increase its sales of combustible cigarettes and influence tobacco control measures in most countries [44].

### Study limitations

This study focused only on newspaper reports and covered only a two-year period. To obtain the whole picture regarding e-cigarettes in Indonesia, studies on other media platforms are needed. More extensive monitoring (before 2020 and after 2021) might also be required, particularly by documenting any activity aimed at promoting e-cigarettes and influencing public health policy.

## Conclusions

The articles and arguments with positive tones toward e-cigarettes outnumbered negative articles and arguments. Among all positive frames toward e-cigarettes, harm reduction was the most dominant frame delivered in the news media. The industry, university, and research-based institutions were the most involved types of organizations in the news articles. Although the health impact is the most reported topic, the actors involved in delivering arguments were mainly from the non-health sector, including when the news articles were published by the newspaper desk. The e-cigarette issue in Indonesia’s news media was mainly played by the non-health sector, which is more inclined toward economic interest than health.

## Data Availability

The datasets used and/or analyzed during the current study are available from the corresponding author on reasonable request.

## Abbreviations

AKVINDO: The Indonesian Vape Consumer Association
APPNINDO: Association of Indonesian Nicotine Delivery Entrepreneurs
APVI: The Indonesia Vapor Entrepreneurs Association
AVI: Indonesia Vapers Association
CYPR: Center for Youth and Population Research
Govt.: Government
INDEF: Institute for Development of Economics and Finance
IYPG: Indonesian Young Pharmacist Group
KABAR: The Indonesia Tar-Free Coalition
KONVO: Organized Vape Consumers
Lakpesdam PBNU: PBNU’s Institute for Research and Human Resources Development
NGOs: Non-Government Organizations
NPPBKC: Excisable Goods Entrepreneur Identification Number
SEATCA: Southeast Asia Tobacco Control Alliance
SNI: The Indonesian National Standard Certification
Univ: University
WHO FCTC: The World Health Organization Framework Convention on Tobacco Control
YPKP: Public Health Observer Foundation
